# Verbal Abuse among Nurses in Tertiary Care Hospitals

**DOI:** 10.31729/jnma.4571

**Published:** 2019-08-31

**Authors:** Kalpana Silwal, Sarala Joshi

**Affiliations:** 1 Chitwan Medical College, Bharatpur, Chitwan, Nepal; 2 Om Health Institute, Nursing College, Kathmandu, Nepal

**Keywords:** *nurses*, *verbal abuse*, *workplace*

## Abstract

**Introduction:**

Verbal abuse against nurses who are major working force can affect the work performance and productivity in hospital. Verbal abuse is epidemic in tertiary care hospitals though it is most preventable with healthcare professions. The study aims to identify the prevalence of verbal abuse among nurses in a Tertiary Care Hospital.

**Methods:**

A descriptive cross-sectional study was conducted among nurses in two tertiary care hospitals in Chitwan using self-administered questionnaire from August–December, 2018. The researcher used the convenient sampling technique and total 331 nurses were taken for the study.

**Results:**

Prevalence of verbal abuse was found to be 122 (36.9 %) [34.25%-39.55% at 95% CI] among nurses. Perpetrators of the verbal abuse were relatives of the patients 64 (48.9%) followed by staff member 29 (23.77%), patient 23 (17.6%), management or supervisor 4 (3.1%) and from colleague 2 (1.5%). Frequency of verbal abuse as sometimes, once and all the times were 95 (77.86%), 20 (16.03%) and 7 (5.7 %) respectively. Study showed that verbal abuse was mostly done by relatives of the patient. Consequence of verbal abuse was disturbing memories, thoughts or an image ultimately reducing the job satisfaction. No any action was taken except verbal warning for 20 (58.8%) perpetrators.

**Conclusions:**

Verbal abuse is frequently prevalent in nurses and mostly from patient's visitors. Hence, nurses should maintain their respect and authority otherwise it will lead to decrease in their performance that shows direct impact on patient care and consequently the effectiveness of the health care system.

## INTRODUCTION

Verbal abuse in nursing is quite costly to the individual nurses, the hospitals and the patients. Nurses who regularly experience verbal abuse may be more stressed, may feel less satisfied with their jobs, miss more work and provide a substandard quality of care to patients. Workplace violence is a serious and common widespread phenomenon.^1^ Nurses face more violence than other professions. Nurses as frontier care providers serve in a wide variety of settings caring for individuals are suffering life-altering events.^2^

Nurses are invaluable members of the organization's work place team.^3^ Specially verbal abuse has a significant impact on the efficiency and effectiveness of healthcare providers.^4^ Nurses are at such a high risk of exposure to violent behaviours.^5^ Consequences of workplace violence affects quality of life.^6^

Therefore, the researcher intends to identify the prevalence of verbal abuse among nurses in tertiary care hospitals.

## METHODS

A descriptive cross-sectional study was done in two tertiary care hospitals, Chitwan Medical College Teaching Hospital and Bharatpur Government Hospital within Chitwan from August to December, 2018. Ethical approval was taken from Nepal Health Research Council (NHRC).

Sample size was calculated as below:

n= Z^2^pq/e^2^

   = (1.96)^2^ × 0.299 × 0.701 / (0.05)^2^

   = 316

where,

n= sample size
p= prevalence of the similar previous study^2^
q= 1-p
e= margin of error, 5%
So, the final sample size is 316.

Using convenient sampling technique, the sample size of 185 and 146 were taken respectively from Chitwan Medical College and Bharatpur Government Hospital respectively. They were included if more than six months experienced in their recent workplace. Researcher's target was all the nurses of these hospitals with minimal of six months of experience. The participation in the study was voluntary. A self-administered structured anonymous questionnaire was used to get information from the participants. Verbal consent was taken before distributing questionnaire.

During the research, 356 respondents were included. Out of 356, only 337 questionnaires were returned among which six of the data sheet were not filled properly so that was discarded, thus 331 questionnaires were valid for analysis. Data was analyzed by SPSS 20 version and descriptive analysis was done to identify prevalence of workplace violence. Statistical analysis was performed to calculate the outcome in frequency and percentage.

## RESULTS

Prevalence of verbal abuse was 112 (36.9%) CI (34.25%-39.55%) among nurses in tertiary care hospitals. Most 95 (77.86%) nurses are abused sometime and nurses who were all the times abused were 7 (5.7%). Most common perpetrators 64 (48.9%) were relatives of the patient and least common 2 (1.5%) perpetrators were colleague (Table 1).

**Table 1:** Distribution of verbal abused in the last 12 months.

Statements	n (%)
Have you ever been verbally abused (Total answered sample =331)	122 (36.9)
How often (Total answered sample = 122)	
All the times	7 (5.7)
Sometimes	95 (77.86)
Once	20 (16.03)
Perpetrators (by Whom), n=122	
Patient/client	23 (17.6)
Relatives of the patient/client	64 (48.9)
Staff member	29 (23.77)
Management /supervisor	4 (3.1)
Colleague	2 (1.5)

Pattern of responses towards verbal abuse is by reporting to seniors which is by 35 (27.3%) and least 5 (4.09%) are sought for counselling. Other responses, on avoiding thinking about or talking about the abuse or avoiding having feelings related to it highest falls on moderately is 56 (45.9%) and least is 17 (13.93%) (Table 2).

**Table 2:** Consequences Pattern after being verbal abused.

Repeated, disturbing memories, thoughts or images of the abuse (Total answered= 122)	
Statement	n (%)
Took no action	22 (17.2)
Tried to pretend it never happened	19 (14.8)
Told the person to stop	21 (16.4)
Told friends/family	9 (7)
Told colleague	11 (8.6)
Report it to senior staff members	35 (27.3)
Sought counseling	5 (4.09)
Avoiding thinking about or talking about the abuse or avoiding having feelings related to it	
**Statement**	**n (%)**
Avoiding thinking about or talking about the abuse or avoiding having feelings related to it, not at all	17 (13.93)
Avoiding thinking about or talking about the abuse or avoiding having feelings related to it, A little bit	30 (24.5)
Avoiding thinking about or talking about the abuse or avoiding having feelings related to it, moderately	56 (45.9)
Avoiding thinking about or talking about the abuse or avoiding having feelings related to it, quiet a bit	19 (15.57)

Patterns of supporting system to report or to investigate is highest, 24 (70.6%) from management or employer and least from community group is 2 (5.9%). Consequences for perpetrators is only verbal warning is for 20 (58.8%) and aggressor prosecuted is only 2 (5.8%). Respondent's satisfaction on handling manner of the incident is only sometimes satisfaction is 11 (32.3)% is higher than others followed by never satisfied 7 (20.5%) as shown (Table 3).

**Table 3:** Action Taken after Being Verbal Abused.

Statement	n (%)
Action taken to investigate the causes of the verbal abuse	
Statement	n (%)
Action was taken	34 (27.86)
Encouraged to take action, from	
Management /employer	24 (70.6)
Union/association	8 (23.5)
Community group	2 (5.9)
Action was taken for Abuser (Consequences for Abuser) Total answered=34	
Statement	n (%)
None	10 (29.6)
Verbal warning	20 (58.8)
Reported to the police	2 (5.8)
Aggressor prosecuted	2 (5.8)
Satisfied with the handled manner (Total answered=34)	
Statement	n (%)
Always satisfied	6 (17.7)
Often satisfied	4 (11.8)
Sometimes	11 (32.3)
Seldom satisfied	6 (17.7)
Never satisfied	7 (20.5)

Respondents’ response not to report for the incident is more in afraid of negative consequences is 15 (44.1%) and least in felt ashamed is 2 (5.9%) as shown (Table 4).

**Table 4:** Reasons not to Report for Workplace Violence.

Statement (Total answered sample=34)	n (%)
It was not important Felt ashamed	7 (20.6%) 2 (5.9%)
Felt guilty	6 (17.6%)
Afraid of negative consequences	15 (44.1%)
Did not know who to report to useless	4 (11.8%)

Most 245 (74.6%) of the respondents were the age of 21- 25 years and least 7 (2.1%) were the age of 31-35 years. Mean age of the respondents was 24.5±5.079 years.

Majority 201 (60.7%) of the respondents were married and 244 (73.8%) were full time workers.

## DISCUSSION

The study shows prevalence of the verbal abuse is 36.9% among the nurses. Studies conducted in one of the tertiary care institution of Nepal and in Baglung district of Nepal found that 40% and 47.7% of health workers were verbally abused.^4,5^ A study showed that verbal abuse was experienced by 87.4% of the population during a 6-month period.^7^ Other study showed that 50 percent registered nurses and nursing students reported being verbally abuse in a 12 month period.^8^ Other studies have reported two-thirds of verbal workplace violence.^9^ The proportion of verbal violence was higher (61.5%).^10^ Different study showed different results so by World Health Organization recommended to record incidents of verbal abuse and threats and evaluate the record on a monthly basis by department safety committee. Reporting systems should be user friendly: easy to understand, not too time consuming and accessible.^4^

This study revealed that 48.9% perpetrators were relatives of the patient or client. A study conducted in Baglung district also showed that the perpetrators of all three types of violence (physical, verbal abuse and sexual harassment) were mostly the relatives of patients. Incidents took place mostly inside the health institution for verbal abuse (84.5%).^4^

Pattern of responses towards verbal abuse by the victims showed that 27.3% told their seniors about the event and only 16.4% of the respondents had told to person to stop for verbal abuse. A study reported that most of the victims of physical violence reported that they told the perpetrators to stop (72.7%) and only 4% had told to their seniors of their verbal abuse. Same study also reported that 3% sought for counselling.^4^ Recent study shows 4.09% sought for counselling, which is similar to findings.

In this study, 44.1% of respondents didn't report for the incident due to fear of negative consequences is which is similar to this study.^5^ In this study, the extent of the repeated, disturbing memories, thoughts or images after the experience of verbal abuse was surprisingly rated as little bit by 36.8% of the respondents. A study showed that psychological consequences resulting from violence may include fear and frustration.^7^ Similar study showed that nurses experienced at least one post-traumatic stress disorder symptom after a violent event by 94%.^6^

Study reports that most (34.4%) of the respondents are moderately “super alert” after being verbal abuse and extremely “super alert” is 4.09%. One of the study showed that health workers always feel stress and hazardous in their workplace after being verbal abuse.^9^ One of the study reported that the effects of abuse on nurses produce sleeping disorders, stress, self dissatisfaction and disappointment.^10^ Nurses suffered from psychological, physical and occupational stress was often due to the workplace violence.^8^ Stress and violence are increasingly noted in health sector workplaces. Doctors, nurses and social workers are all high on the list of occupations with serious stress levels, while violence in the health sector constitutes almost a quarter of all violence at work. The enormous cost of work stress and violence at work for the individual, the workplace and the community at large is becoming more and more apparent.^11^ Negative stress as a source of violence has been identified for several occupations. Negative stress activates a variety of physical and emotional symptoms that can lead to serious illness.^12^

This study shows consequences for perpetrators is only verbal warning accounting 58.8% and 5.8% of the aggressors prosecuted. Likewise patterns of supporting system to report or to investigate is highest 70.6% from management or employer and least 5.9% from community group. In the US 100% of respondents witnessed verbal violence, whereas only 23% of verbal abuse cases were reported.^9^ In India, a study showed that verbal abuse witnessed was 89% but the reported cases was only from 46%.^7^ Today, there is increased evidence that health staff especially nursing staffs are at such a high risk of exposure to violent behaviours in the workplace; it is now considered to be a major occupational hazard worldwide.^11^

The study was limited to only two tertiary level hospitals. It was limited only on structured questionnaire and verbal abuse might be among other healthcare stakeholders but this study is only limited within Nurses only.

## CONCLUSIONS

Verbal abuse is frequently prevalent in nurses and mostly from patient's or client's visitors. Hence, nurses should maintain respected and authorized place if not it will lead to decrease in performance which will have direct impact on patient care and consequently the effectiveness of the health care system.
